# Altered Intranetwork and Internetwork Functional Connectivity in Type 2 Diabetes Mellitus With and Without Cognitive Impairment

**DOI:** 10.1038/srep32980

**Published:** 2016-09-13

**Authors:** Shi-Qi Yang, Zhi-Peng Xu, Ying Xiong, Ya-Feng Zhan, Lin-Ying Guo, Shun Zhang, Ri-Feng Jiang, Yi-Hao Yao, Yuan-Yuan Qin, Jian-Zhi Wang, Yong Liu, Wen-Zhen Zhu

**Affiliations:** 1 Department of Radiology, Tongji Hospital, Tongji Medical College, Huazhong University of Science and Technology, Wuhan, Hubei, 430030, China; 2 Department of Pathophysiology, School of Basic Medicine and the Collaborative Innovation Center for Brain Science, Key Laboratory of Neurological Diseases of Education Ministry of China, Tongji Medical College, Huazhong University of Science and Technology, Wuhan, Hubei, 430030, China; 3 Department of Neurology, Wuhan General Hospital of Guangzhou Command, Wuhan, Hubei, 430070, China; 4 School of Biomedical Engineering, Southern Medical University, Guangzhou, Guangdong, 510515, China; 5 Brainnetome Center, Institute of Automation, Chinese Academy of Sciences, Beijing, 100190, China; 6 National Laboratory of Pattern Recognition, Institute of Automation, Chinese Academy of Sciences, Beijing, 100190, China

## Abstract

Type 2 diabetes mellitus (T2DM) is associated with cognitive impairment. We investigated whether alterations of intranetwork and internetwork functional connectivity with T2DM progression exist, by using resting-state functional MRI. MRI data were analysed from 19 T2DM patients with normal cognition (DMCN) and 19 T2DM patients with cognitive impairment (DMCI), 19 healthy controls (HC). Functional connectivity among 36 previously well-defined brain regions which consisted of 5 resting-state network (RSN) systems [default mode network (DMN), dorsal attention network (DAN), control network (CON), salience network (SAL) and sensorimotor network (SMN)] was investigated at 3 levels (integrity, network and connectivity). Impaired intranetwork and internetwork connectivity were found in T2DM, especially in DMCI, on the basis of the three levels of analysis. The bilateral posterior cerebellum, the right insula, the DMN and the CON were mainly involved in these changes. The functional connectivity strength of specific brain architectures in T2DM was found to be associated with haemoglobin A1c (HbA1c), cognitive score and illness duration. These network alterations in intergroup differences, which were associated with brain functional impairment due to T2DM, indicate that network organizations might be potential biomarkers for predicting the clinical progression, evaluating the cognitive impairment, and further understanding the pathophysiology of T2DM.

Type 2 diabetes mellitus (T2DM) is one of the most common diseases in elderly people. Neurodegeneration can occur because of the accumulation of advanced glycation end products (AGEs), which can lead to inflammation, oxidative stress, protein cross-linking, cerebral insulin resistance and metabolic disorders^1^. Moreover, D-ribose is an efficient glycator and is much more active in protein glycation than D-glucose under identical conditions, D-ribose, not D-glucose, rapidly reacts with proteins and produces significant amounts of AGEs, which may impair spatial cognition, similarly to how ribosylation triggers Alzheimer’s disease-like Tau hyperphosphorylation^2^. T2DM, a common metabolic disease, increases the risk for cognitive impairment, cognitive decline and dementia^3^,^4^,^5^,^6^,^7^. Recent neuroimaging studies have demonstrated that the structural and functional alterations may provide potential biomarkers for predicting future cognitive performance. Briefly, T2DM, as compared with the absence of diabetes, is associated with grey matter volume loss^8^,^9^, reduced cortical thickness thinning^10^, microstructural white matter abnormalities^11^, cerebral white matter network disruption^11^,^12^, and impaired spontaneous brain activity^13^,^14^. Although these abnormalities are not specifically correlated with distinct features, they are clearly associated with cognitive performance in patients with T2DM^5^,^6^,^15^.

Awareness that patients with T2DM are not only weak in one specific daily function but also perform poorly in broad aspects of daily cognition, such as memory, attention, and executive function, is increasing; there is a higher risk of information-processing speed reduction in mild cognitive impairment and dementia^3^,^7^,^14^. In fact, the human brain is organized to be an effective functional system in which segregated complex systems with different functional areas are not only specialized for processing distinct forms of information but also exchange information at a very high speed^16^,^17^. The network/connectivity model is **a** simple but powerful tool that can provide new insights into understanding the general principle of brain functions and abnormalities in brain disorders^18^,^19^. Consequently, evaluating the functional disturbances across different brain networks is very important for understanding the cognitive impairment in T2DM patients. Fortunately, increasing numbers of studies have observed impaired connectivity of the hippocampus, default mode network (DMN) and other brain systems associated with cognitive decline in patients with T2DM^20^,^21^,^22^,^23^,^24^. However, the pathophysiology underlying the cognitive ability and brain connectivity changes in patients with T2DM is not well understood for many of the following reasons: 1) most of the previous studies have focused only on comparison between T2DM and non-diabetic controls, and 2) previous studies have mainly focused on specific brain regions or specific brain networks.

From the earlier findings in people at risk for AD^25^,^26^,^27^, we hypothesized that, compared with normal control subjects, T2DM patients especially those with cognitive impairment, would be associated with impaired functional connectivity in the five key networks; furthermore this impaired functional connectivity would be associated with cognitive ability. To test this hypothesis, we evaluated the functional connectivity patterns at three levels (integrity, network and connectivity) within five key RSNs: the default mode network (DMN), the dorsal attention network (DAN), the control network (CON), the salience network (SAL) and the sensorimotor network (SMN), on the basis of the resting-fMRI data acquired from 19 T2DM patients with cognitive impairment (DMCI), 19 T2DM patients with normal cognition (DMCN) and 19 well matched healthy controls (HC) with normal cognition. Additionally, we investigated the relationships between the clinical scores (the Mini Mental State Examination (MMSE), haemoglobin A1c (HbA1c) and the duration of T2DM) and the strength of functional connectivity at each scale using Pearson’s correlations after controlling for age, gender and education effects in the patient groups (Fig. 1).

## Materials and Methods

### Participants

This cross-sectional study followed the Declaration of Helsinki tenets and was approved by the Institutional Review Board (IRB) of the performing hospital. Informed consent forms were signed by all of the subjects after a full description of the MRI examination in the study was provided to them. The methods were carried out in accordance with the approved guidelines.

Laboratory examinations such as plasma glucose and HbA1c assays were performed. The Hachiski ischaemic score (HIS) was used to exclude vascular dementia (VD). All of the subjects underwent physical, neurological, and neuropsychological assessments such as MMSE, Montreal Cognitive Assessment (MoCA), and the Activity of Daily Living (ADL) score.

Sixty-four right-handed subjects were recruited to participate in this study. According to the American Diabetes Association recommendation and prior diagnostic criteria^28^, the subjects were divided into HC and T2DM groups. The HC group inclusion criteria consisted of the following: (1) no symptoms of diabetes; (2) fasting plasma glucose < 7.0 mmol/L; (3) HbA1c < 6.0%; (4) Mini-Mental State Examination (MMSE) scores ≥ 27; and (5) Montreal Cognitive Assessment (MoCA) cores ≥ 26. The HC group exclusion criteria consisted of the following: (1) contraindication for MRI scanning; (2) evidence of abnormalities in the brain; (3) evidence of memory decline; (4) any types of systemic diseases; and (5) any psychiatric diseases. The T2DM group inclusion criteria consisted of the following: (1) symptoms of diabetes; (2) history of 2 type diabetes mellitus; (3) fasting plasma glucose ≥ 7. 0 mm ol/L; (4) random plasma glucose ≥ 11. 1 mmol/L; and (5) 2-h plasma glucose ≥ 11. 1 mmol/L after oral glucose tolerance test; and (6) HbA1c ≥ 6.5%. The T2DM group exclusion criteria consisted of the following: (1) contraindication for MRI scanning; (2) evidence of abnormalities in the brain; and (3) any other types of systemic diseases. Then, the T2DM subjects were subdivided into DMCI and DMCN groups. The inclusion criteria for the DMCI group were: (1) evidence of memory decline that could affect maintaining normal daily activities; (2) MoCA and MMSE scores of ≤ 27 points; and (3) lack of any other physical or mental disorders that could lead to abnormal cognition.

After quality control (i.e., age, gender balance and image quality control), eleven females and 8 males (mean age 60.21, range from 52 to 76 years old) were included in the HC group, 12 females and 7 males (mean age 59.53, range from 51 to 72 years old) were included in the DMCN group and 14 females and 5 males (mean age 61.95, range from 52 to 70 years old) were included in the DMCI group. The three groups of subjects were age, gender and education-matched without any intergroup significant differences (*P* > 0.05). The group specific demographics, neuropsychological and clinical characteristics are provided in Table 1.

### MRI acquisition

All of the subjects underwent MRI scanning in Tongji Hospital, Wuhan, Hubei, China. A 3.0 Tesla scanner (Discovery MR750, GE Healthcare, Milwaukee, WI, USA) with 32-channel head array coil was used in this study. All of the subjects were able to fully communicate and were instructed to relax their minds and keep their eyes closed while being awake during scanning. All of the subjects were scanned in a supine and head-first position. Gradient echo - echo planar imaging (GRE-EPI) sequences were applied to scan with the following acquisition parameters: repetition time (TR)/echo time (TE) = 2000/35 ms, field of view (FOV) = 240 mm × 240 mm, matrix = 64 × 64, flip angle (FA) = 90°, axial slices = 40, slice thickness = 4 mm with no gap, scan time = 8 min, 240 volumes for each subject. Structural 3D-BRAVO sequences were scanned with: TR/TE/inversion time (TI) = 8.2/3.2/450 ms, FOV = 256 mm × 256 mm, slice thickness = 1 mm, sagittal slices = 166, acquisition matrix = 256 × 256, number of signal averages (NEX) = 1, FA = 12°, bandwidth = 31.25 Hz, scan time = 4 min 22 s. Additionally, routine T2 weighted MRI scanning was performed by two radiologists to exclude organic disease of the brain. The lights of the scanner and the scanning room were turned off to minimize the interference while scanning. Conditions such as no head motion were ensured by patient instructions and symmetrical cushions that were comfortably placed.

### Data preprocessing

Standard in-house Brainnetome fMRI toolkit (Brant, http://www.brainnetome.org/en/brainnetometool.html) based on statistical parametric mapping (SPM8, http://www.fil.ion.ucl.ac.uk/spm) was applied for data preprocessing and analysis. The steps of preprocessing consisted of the following: (1) converting DICOM files to NIFTI images, (2) slice timing after discarding the first 10 time points, (3) realignment to the first volume, (4) normalization to a standard EPI template and reslicing to 2 mm isotropic voxels, (5) de-noising by regressing out several effects including six head-motion parameters, constant, linear drift, and mean time series of the voxels within the white matter and cerebrospinal fluid mask, (6) band pass temporal filtering (0.01–0.08 Hz), and (7) smoothing by 6 mm full width at half maximum (FWHM) Gaussian kernel.

### Functional connectivity analysis

The functional connectivity patterns of the five key RSNs (DMN, DAN, CON, SAL and SMN) based on 36 previously defined regions of interest (ROIs) have been well studied previously^25^,^26^,^27^,^29^,^30^,^31^. For each of these 36 previously defined ROIs, spheres with 6 mm radii were drawn to represent the five RSNs. To maintain the integrity of the present paper, the Montreal Neurological Institute (MNI) coordinates of the 36 ROIs, which are the same as in Brier and colleagues’ study, are provided in Table S1.

In each ROI, the time series of all of the voxels were averaged to obtain the mean time series to represent the activity of the brain region; i.e., 36 (ROI number) × 230 (time points) were obtained for each subject. Pearson’s correlation coefficients (*r* values) were calculated between the mean time series of each pair of regions. The correlation coefficients were converted to η values by using an exponential function related to the connectivity “distance” between the two connected ROIs, **i.e.**, 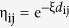, where 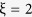
32,33 and 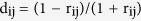 is a hyperbolic correlation measure denoting the distance between two nodes (in this case, the network node is the brain region of interest), where r_ij_ is Pearson’s correlation coefficient. We obtained three measures at three levels of analysis:

1. Nodal integration: the total degree of node i was calculated using 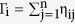 as the measurement of nodal integration, i.e., each node’s connectivity with the rest of the brain^33^.
2. Network level: the intranetwork strength was defined as the mean connection strength of the ROIs in the same network for each of the five RSNs: 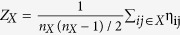, where n_*X*_ represents the number of ROIs within a specific subnetwork X; internetwork connectivity strength was defined as the mean connection strength of all of the possible connections for each subnetworks-pair: 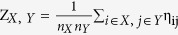, where X and Y represent the subnetworks of the five RSNs^25^.
3. Large scale connectivity: large scale connectivity represents the η_ij_ scores of each ROIs-pair among the prior 36 seed ROIs^27^.

### Statistical analysis

One-way Analysis of Variance (ANOVA) was used to evaluate the statistical significance (*P* < 0.05, corrected for multiple hypothesis testing by using a permutation based method at *P* < 0.05) of the Z scores at each level among 3 groups (HC, DMCN and DMCI) after controlling for age, gender and education effects by using a general linear model. For all of the identified connectivity, *post hoc* analysis based on two-sample two-sided *t*-tests were used to assess the significant effects of the HC versus DMCN, HC versus DMCI and DMCN versus DMCI (*P* < 0.05).

In the T2DM (DMCN and DMCI) subjects, Pearson’s correlations between HbA1c, T2DM illness duration and the Z scores were explored to investigate the relationship between the functional connectivity and the clinical measures of diabetes at each level. In the DMCN and DMCI groups, Pearson’s correlations between the MMSE scores and the Z scores were also calculated to investigate the relationship between the functional connectivity and the cognitive ability. The statistical threshold was set at *P* < 0.05 for exploratory purposes.

## Results

### Clinical and neuropsychological data

T2DM has been identified as an independent risk factor for cognitive impairment or dementia. In the present cross-sectional study, we collected the clinical index and cognitive scores of subjects, such as the illness duration, the HbA1c level, the fasting glucose level, the MMSE scores and the MoCA scores. The demographic characteristics and neuropsychological data for each group are presented in Table 1. No significant differences were observed among groups with respect to age, gender and education level. As expected, the T2DM patients had higher levels of HbA1c and fasting glucose (all *P* < 0.001) than HC. In terms of cognitive performance, DMCI patients had poorer scores measured with MMSE and MoCA than DMCN and HC subjects (all *P* < 0.001). In the DMCI group, HbA1c showed a significant correlation with the illness duration (*r* = 0.634, *P* = 0.008) after controlling for age, gender and education effects. The results indicated that the T2DM patients had higher HbA1c and fasting glucose levels than the HC group, especially the DMCI patients. Moreover, the HbA1c level increased as the illness duration increased in the DMCI group.

### Differences among the three groups

**Figure 1:**
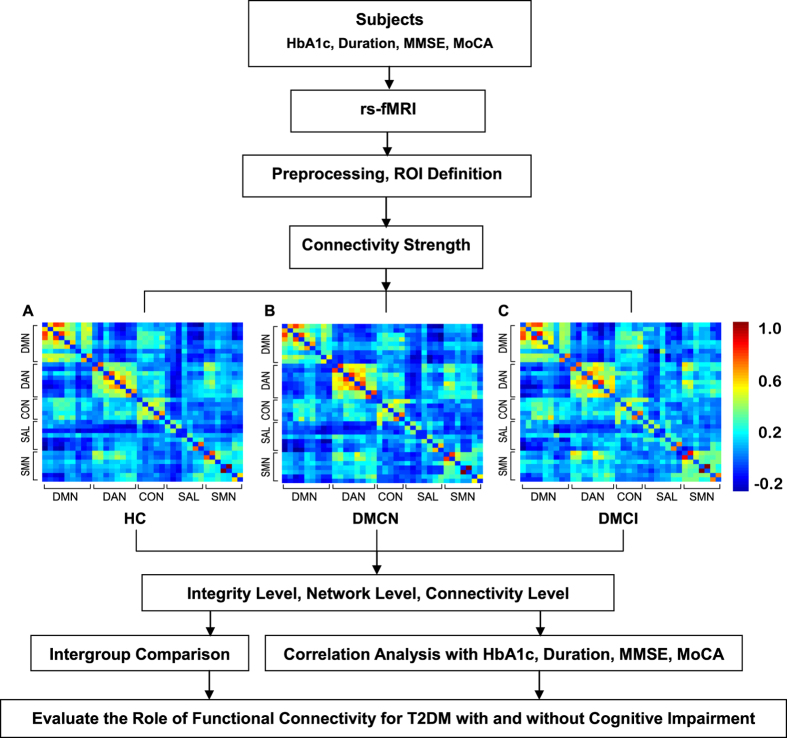
Experiment schematic figure. (**A**–**C**) An overview of the group mean connectivity among 36 ROIs, which consisted of 5 key resting-state networks (RSNs) in the HC, DMCN and DMCI groups.

For each group, a 36 × 36 functional connectivity matrix was determined by computing Pearson’s correlation coefficients of the mean time series of each ROI (Fig. 1). In the HC group, the majority of strong positive functional connectivity was within each subnetwork. A similar pattern was observed in the DMCN and DMCI groups, except that the intranetwork and internetwork connectivity was reduced. We carried out ANOVA statistics at three levels (integrity, network and connectivity) to investigate the altered connectivity pattern among the HC, MDCN and DMCI groups.

**Figure 2:**
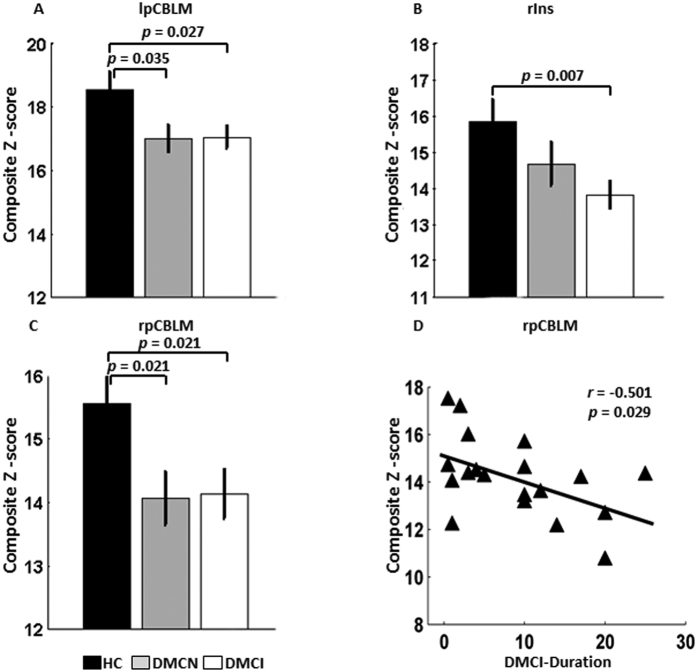
Intergroup differences and clinical correlation at the integrity level. Compared with the HC group, (**A**) decreased connectivity strength of the lpCBLM is found across the DMCN and DMCI groups, (**B**) decreased connectivity strength of the rIns is found in the DMCI group, (**C**) decreased connectivity strength of the rpCBLM is found across the DMCN and DMCI groups, (**D**) the connectivity strength of the rpCBLM in the DMCI group is negatively correlated to the illness duration.

At the integrity level, significant group differences were anchored in the bilateral posterior cerebellum (pCBLM) and the right insula (rIns) (Fig. 2). *Post hoc* analysis showed that the integrity connectivity of the bilateral pCBLM was decreased in the DMCN and DMCI groups compared with the HC group. Significantly decreased connectivity strength of the rIns was found in the DMCI group compared with the HC group; however, there were no significant alterations between the DMCN and DMCI groups (Table 2, Fig. 2). The pCBLM is an important part of the DMN; the rIns is a key region of the SAL. From the above results, we concluded that the altered connectivity related to the DMN and SAL was involved in the T2DM patients at the integrity level. Moreover, it is important to note the decreased connectivity strength of the pCBLM in the DMCN and DMCI groups.

**Figure 3:**
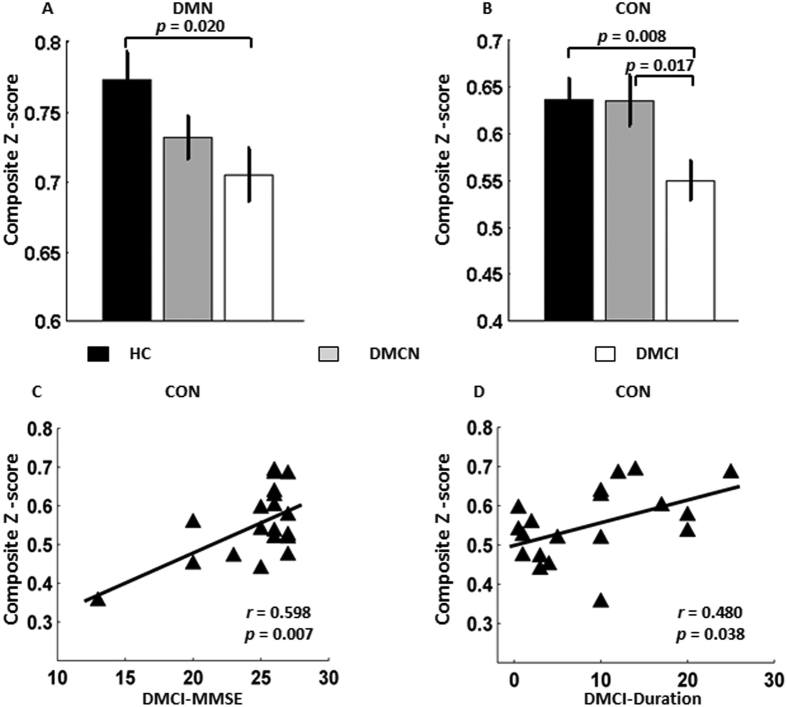
Intergroup differences and clinical correlations at the network level. Compared with the HC group, (**A**) decreased connectivity strength within the DMN is found in the DMCI group. Compared with the HC and DMCN groups, (**B**) there are decreased connectivity strength within the CON in the DMCI group. (**C**) A positive correlation between the connectivity strength within the CON and MMSE scores is found in the DMCI group. (**D**) A positive correlation between the connectivity strength within the CON and the illness duration is found in the DMCI group.

As shown in Fig. 3, the Z scores of the DMN and the CON showed significant differences among the three groups. Significantly impaired intranetwork connectivity within the DMN (*P* = 0.020) and CON (*P* = 0.008) was found in the DMCI patients but not in the DMCN patients compared with the HC group; significantly impaired intranetwork within the CON (*P* = 0.017) was found in the DMCI patients compared with the DMCN group (Table 2, Fig. 3). No significantly impaired internetwork connectivity was found among the 3 groups. The altered intranetwork connectivity strength was found within the DMN and the CON in the T2DM patients. Notably, the decreased intranetwork connectivity strength within the CON in the DMCI group compared with the DMCN group helped us to distinguish the DMCI patients from the DMCN group effectively.

**Figure 4:**
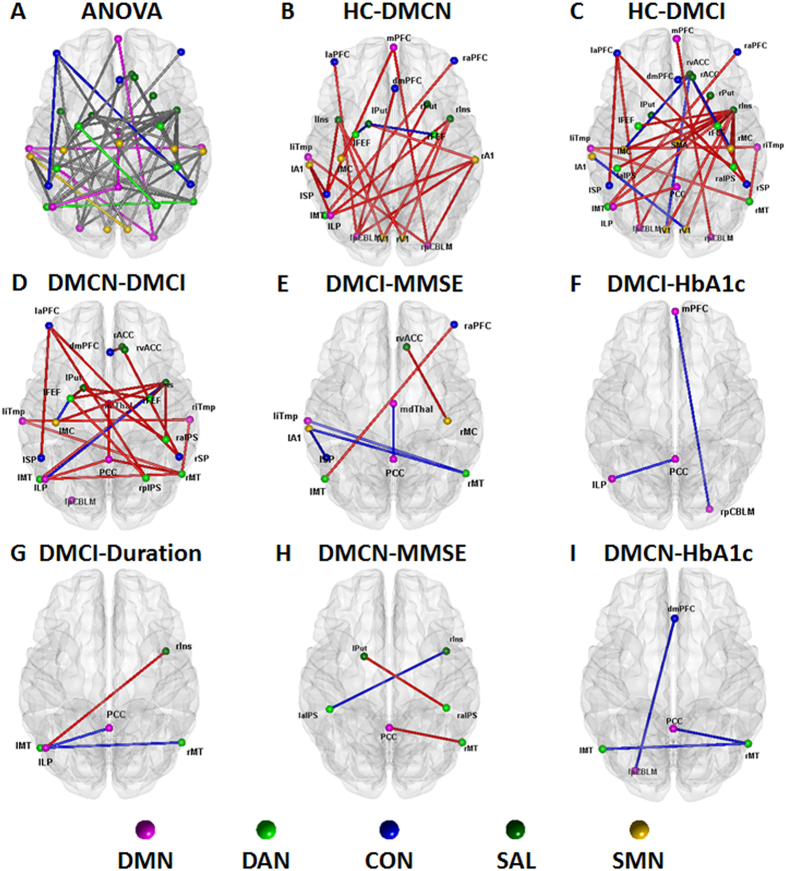
Connectivity pairs with significant differences and clinical correlations at the connectivity level. (**A**) Topological mapping of 50 intranetwork and internetwork connectivity pairs with significant differences among the HC, DMCN and DMCI groups (ANOVA, permutation corrected *P* < 0.05); node with different color means different subnetwork, grey edge means internetwork connectivity, other edges mean intranetwork connectivity. (**B**–**D**) Topological mapping of the connectivity pairs with significant differences between HC and DMCN, HC and DMCI, DMCN and DMCI; red edges mean that the connectivity strength of the former group is higher than the latter, blue edges mean converse. (**E**) Four negative and 2 positive correlations between the connectivity strength and MMSE scores in the DMCI group, (**F**) 2 negative correlations between the connectivity strength and HbA1c in the DMCI group, (**G**) 2 negative and 1 positive correlations between the connectivity strength and illness duration in the DMCI group, (**H**) 1 negative and 2 positive correlations between the connectivity strength and MMSE scores in the DMCN group, (**I**) 3 negative correlations between the connectivity strength and HbA1c level in the DMCN group; red line represents positive correlation, blue line represents negative correlation.

At the connectivity level, fifty changed intranetwork and internetwork functional connectivity pairs were identified among the three groups by one-way ANOVA (all *P* < 0.05) after controlling for the effects of age, gender and education (Fig. 4A, and Table S4). These 50 connectivity pairs were widely distributed, mainly in the internetwork connectivity between the SMN and SAL and between the DMN and other RSNs (Fig. 4A). *Post hoc* analysis showed that the DMCI subjects were associated with more impaired connectivity (Fig. 4B–D, Table S4). Among the nodes connected with these impaired pairs, the most affected region was the rIns (Fig. 4A–D, Table S4). Consistently with the results from previous imaging studies^13^,^14^,^34^, our results suggested that abnormal functional connectivity existed in T2DM patients.

### Correlations between the altered connectivity and clinical information

Because clinical information may be associated with cognitive ability in T2DM patients, we carried out correlation analyses at three levels (integrity, network and connectivity). At the integrity level, a longer disease duration was significantly associated with lower functional connectivity strength of the rpCBLM in the DMCI group (*r* = −0.501, *P* = 0.029) (Table 2, Fig. 2); no other significant correlation was found. The connectivity strength of the rpCBLM, which belongs to the DMN, decreased with increasing illness duration. At the network level, within the CON in the DMCI group, decreased cognition ability (lower MMSE) was significantly correlated with a greater loss of intranetwork connectivity (*r* = 0.598, *P* = 0.007). Moreover, we found that the connectivity strength showed a positive correlation with the illness duration within the CON in the DMCI subjects (*r* = 0.480, *P* = 0.038) and a slight negative correlation with MoCA scores within the CON in the DMCN subjects (*r* = −0.475, *P* = 0.040) (Table 2, Fig. 3). The results suggested that the CON may play important and complex roles in cognitive impairment and compensatory mechanisms in T2DM patients at the network level.

As shown in Fig. 4, six pairs of connectivity were significantly (2 positive and 4 negative) associated with the MMSE scores in the DMCI group, whereas 3 pairs (2 positive and 1 negative) of connectivity were significantly correlated with the MMSE scores in the DMCN group (Fig. 4E,H, Table 3; for details, please refer to Table S4). Our results also demonstrated that 2 connectivity pairs showed negative correlations with HbA1c in the DMCI group and 3 connectivity pairs showed negative correlations with the HbA1c level in the DMCN subjects in the identified impaired connectivity pairs (Fig. 4F,I, Table 3; for details please refer to Table S4). In addition, 2 connectivity pairs showed negative correlations with the illness duration, whereas 1 connectivity pair showed positive correlations with the illness duration in the DMCI group in the identified impaired connectivity pairs (Fig. 4G, Table 3; for details please refer to Table S4). Among these significant correlations with the clinical information at the connectivity level, we found that the PCC is the most important region involved in the connectivity pairs. It is well known that the PCC is a key region of the DMN; the DMN is closely involved with episodic memory processing^35^ because the DMN is the most important resting state network. Therefore, the results also suggested that the DMN was closely associated with the cognitive impairment in the T2DM patients.

## Discussion

The main contribution of the present study is that we studied the impaired connectivity patterns in clinical T2DM subjects with and without cognitive impairment. Network/connectivity analysis of both the internetwork and the ROI pairs identified widespread impaired connectivity in T2DM subjects, especially in the DMCI individuals. Another novelty of the present study is that part of the identified impaired connectivity demonstrated significant correlations with cognitive ability as assessed by the MMSE scores; clinical measures such as HbA1c and illness duration (Figs 2, 3, 4) suggest that the cognitive dysfunction might be an effect that is exacerbated by hyperglycaemia.

The present results showed that the patients with T2DM perform slightly worse on a range of cognitive tasks. In terms of MMSE, MoCA and ADL performance, patients with T2DM, on average, performed 0.3–0.4 standard deviations lower than people without diabetes (HC group), which was in line with the results from many previous large-scale epidemiological and meta-analysis studies^6^,^7^,^36^. This finding is also supported by a recent longitudinal study that has shown that patients with T2DM have a decreased ability to regulate blood flow to the brain when needed, and have lower performance in thinking and memory tests^37^.

### Impaired functional connectivity associated with the DMN

The present study shows that the functional connectivity within the DMN (Figs 2, 3) and between the DMN and other regions (Fig. 4A) is significantly impaired in patients with T2DM, especially in DMCI subjects. Because the DMN is the most active brain system in the resting-state, it is closely associated with internal recognition, such as episodic memory, theory of mind and self-evaluation^38^,^39^. A dynamic equilibrium of interaction within the DMN and between the DMN and other brain systems is very important to maintain the normal cognitive function^40^. Several previous studies have demonstrated that the DMN is one of the most affected networks in AD^35^,^41^ and MCI^27^,^42^ subjects. The T2DM population has a high risk of cognition dysfunction, and patients with T2DM show reduced functional connectivity^22^,^23^ and abnormal glucose metabolism^43^ in the DMN. The current study also reveals that the regional connectivity of the bilateral posterior cerebellum, which is one of the representative regions of the DMN, shows duration-associated reduced connectivity strength; together with the observation that the connectivity strength of rpCBLM-mPFC showed significant negative correlation with HbA1c in the DMCI (Fig. 4F), these results indicate that hyperglycaemia contributes to brain abnormalities in subjects with T2DM. Importantly, it should be emphasized that the posterior cingulate cortex (PCC), one of the key regions of the DMN, has been identified as the most involved region in evaluation of the relationship between the impaired connectivity and the clinical or cognitive measures in the T2DM subjects (Fig. 4E–I). This finding is supported by previous studies reporting that aberrant functional connectivity of the PCC to selected brain regions is associated with lower fractional anisotropy of the white matter in the cingulum bundle and uncinated fasciculus^21^ and also with insulin resistance^44^ in patients with T2DM. We found that the connectivity strength of bilateral pCBLM in the DMCN and DMCI groups was significantly decreased compared with that in the HC group. Our findings, in conjunction with other studies consistently suggest that the activity/interaction of the DMN may play a central and fundamental role in cognitive decline in patients with T2DM.

### Impaired functional connectivity associated with the CON

Another novel finding in this study is the abnormal functional connectivity with the CON in the DMCI group compared with the HC and DMCN groups (Fig. 3). The CON is related to executive function and is crucial for high level cognitive functions such as planning, decision making, and the control of attention and working memory^45^,^46^. Recent network studies have also shown impaired connectivity pattern in CON in the MCI subjects^25^,^27^,^47^. In addition, as mentioned elsewhere, T2DM is a well-recognized risk factor for dementia and cognitive decline, so it is unsurprising that the DMCI individuals showed decreased cognitive ability and associated decreased connectivity within the CON (Fig. 3B,C). It is very interesting that only the DMCI group but not the DMCN group had significantly decreased functional connectivity within the CON compared with that in the HC group. Furthermore, network organizations might be an important biomarker to distinguish the DMCI patients from the DMCN patients. However, we also note that the illness duration had a weak positive correlation (*r* = 0.480, *P* = 0.038) with the connectivity strength within the CON in the DMCI group. This result might reflect the complex effects in patients in the DMCI group. One possible reason is that T2DM with mild cognitive impairment requires a compensatory recruitment of cognitive regions while daily activities are performed. This result also indicates that additional studies involving more people and extending for a longer time period are needed to better understand the relationships among the impaired network architecture, changes in cognitive ability and duration of illness. The CON plays an important and complex role in the DMCI group.

### Further discussion, limitation and future directions

We provided convergent evidence that the right insula is the most affected region in the DMCI group and has impaired functional connectivity (Fig. 4A,D). Convergent evidence suggests that the insula is one of the key regions of the salience network, which unites the salient cognitive, conflict monitoring and emotional or homeostatic centres^48^,^49^. Our results strengthen the previous findings that the interactions among the CON, SAL and DMN are important for cognitive control and are impaired in subjects with mild cognitive impairment^27^.

The human brain consists of multiple distinct and interacting networks; investigation of these networks has provided a systematic framework for understanding fundamental aspects of human brain organization and function. The present study only investigates the connectivity patterns on the basis of 36 previously defined regions that represent 5 key RSNs and disregards some important regions such as the hippocampus. Therefore, more finely defined whole brain parcellation schemes based on fine spatial scales^50^ are necessary for future research. Moreover, we evaluated only the first order linear relationship between the network properties and the clinical scores and ignored the complex interaction effect or the higher order correlation, based on the relative small sampling size and cross-sectional design; therefore, we lost dynamic information for evaluating the T2DM effect on cognition. Further intranetwork and internetwork changes may possibly indicate disease progression. Thus, additional longitudinal large sample studies are required. Finally, regarding the CON at the network level, the complex effects of the positive correlation with DMCI-Duration and the slight negative correlation with DMCN-MoCA might be involved in a compensatory mechanism in cognitive impairment in T2DM patients; these topics will require further research in the future.

## Conclusion

This present cross-sectional study sought to reveal the altered resting-state functional connectivity in DMCN and DMCI patients. The changes in connectivity strength in T2DM, especially in DMCI patients, compared with the HC group, indicates that widespread alterations are associated with cognitive impairment. In addition to the effects of the DMN and the SAL, important and complex effects of the CON were found to be associated with the progression of T2DM and to cause cognitive impairment. The T2DM subjects with cognitive impairment manifest chronic hyperglycaemia or increase of HbA1c. Decrements in cognitive function in subjects with T2DM have been found to be correlated with increased duration of diabetes and poor glycaemic control. The alterations of the connectivity strength and their correlations with clinical measures indicate that network organizations may serve as potential biomarkers in predicting the clinical progression of T2DM causing cognitive impairment.

## Additional Information

**How to cite this article**: Yang, S. Q. *et al*. Altered Intranetwork and Internetwork Functional Connectivity in Type 2 Diabetes Mellitus With and Without Cognitive Impairment. *Sci. Rep*. **6**, 32980; doi: 10.1038/srep32980 (2016).

## Supplementary Material

Supplementary Information

## Figures and Tables

**Table 1:** Demographic information for subjects in each group.

	HC (n = 19)	DMCN (n = 19)	DMCI (n = 19)	*F*/*χ2* value	*P* value
Gender (female: male)	11:8	12:7	14:5	1.078	0.583^*^
Age (years)	60.21 ± 5.35	59.53 ± 6.17	61.95 ± 5.93	0.872	0.424^†^
Education (years)	10.68 ± 2.75	11.95 ± 3.21	10.58 ± 3.06	1.215	0.305^†^
diabetes duration (years)	—	5.64 ± 4.57	8.84 ± 7.58	—	0.058^‡^
HbA1c (%)	5.37 ± 0.35	6.98 ± 1.32^a^	8.31 ± 1.53^a,b^	29.263	<0.001^†^
Fasting glucose (mmol/L)	5.16 ± 0.48	10.20 ± 3.22^a^	10.37 ± 2.05^a^	33.656	<0.001^†^
Postprandial glucose	—	14.97 ± 4.42	14.56 ± 4.91	—	0.788^‡^
MMSE	28.74 ± 1.05	28.63 ± 0.96	24.63 ± 3.51^a,b^	21.738	<0.001^†^
MoCA	27.53 ± 0.91	28.21 ± 0.63	24.63 ± 3.10^a,b^	19.058	<0.001^†^
ADL	100 ± 0.00	99.31 ± 2.98	99.74 ± 1.15	0.665	0.518^†^
HIS	1.05 ± 0.62	2.05 ± 1.13^a^	2.11 ± 0.99^a^	7.570	0.001^†^

Data are mean ± standard deviation or number (%) unless otherwise indicated. ^*^The p value was obtained using a Pearson Chi-square test (2-sided). ^†^The p value was obtained using an Analysis of Variance test (ANOVA). ^‡^The p value was obtained using a 2-Tailed t-test between DMCN and DMCI groups. ^a^Significant compared to HC. ^b^Significant compared to DMCN.

**Table 2:** Intergroup differences and clinical correlations at the integrity and network levels.

Level								Correlation					
	ANOVA							Duration (DMCI)		MMSE (DMCI)		MoCA (DMCN)	
	region	HC-DMCN	HC-DMCI	DMCN-DMCI	*F*	*P*	*P*_*permutation*_	*r*	*P*	*r*	*P*	*r*	*P*
integrity	lpCBLM	**0.035**	**0.027**	0.950	3.791	**0.029**	0.029	−0.359	0.131	−0.093	0.704	0.224	0.357
	rpCBLM	**0.021**	**0.021**	0.911	4.036	**0.023**	0.024	**−0.501**	**0.029**	−0.223	0.360	−0.052	0.833
	rIns	0.179	**0.007**	0.236	3.580	**0.035**	0.035	0.341	0.153	0.097	0.691	−0.171	0.484
intranetwork	DMN	0.116	**0.020**	0.280	3.465	**0.038**	0.042	−0.404	0.086	0.251	0.300	0.101	0.681
	CON	0.982	**0.008**	0.017	4.406	**0.017**	0.017	**0.480**	**0.038**	**0.598**	**0.007**	**−0.475**	**0.040**

Abbreviations: lpCBLM: left posterior cerebellum; rpCBLM: right posterior cerebellum; rIns: right insula; CON: control network; DMN: default mode network.

**Table 3:** Correlations between connectivity strength and clinical index at the connectivity level.

	Pair	*r*	*p*
DMCN-MMSE	PCC-rMT	0.510	0.026
	raIPS-lPut	0.501	0.029
	laIPS-rIns	−0.474	0.040
DMCI-MMSE	lSP-lA1	−0.693	0.001
	rMT-lA1	−0.519	0.023
	PCC-mdThal	−0.503	0.028
	rvACC-rMC	0.478	0.039
	liTmp-rMT	−0.477	0.039
	lMT-raPFC	0.459	0.048
DMCN-MoCA	rvACC-rMC	0.463	0.046
	raIPS-rIns	−0.459	0.048
DMCI-MoCA	lLP-lA1	−0.505	0.027
	rV1-lA1	0.482	0.036
	liTmp-rMT	−0.466	0.045
DMCN-HbA1c	lMT-rMT	−0.514	0.024
	lpCBLM-dmPFC	−0.477	0.039
	PCC-rMT	−0.460	0.048
DMCI-HbA1c	mPFC-rpCBLM	−0.506	0.027
	PCC-lLP	−0.501	0.029
DMCI-Duration	PCC-lLP	−0.529	0.020
	lMT-rMT	−0.521	0.022
	lLP-rIns	0.480	0.038

See Table S1 for the abbreviations and coordinates of regions.
