# Supplementary material for: Altered Intranetwork and Internetwork Functional Connectivity in Type 2 Diabetes Mellitus With and Without Cognitive Impairment
Source: Sci Rep. 2016 Sep 13;6:32980. doi: 10.1038/srep32980 (PMC5020685; doi:10.1038/srep32980)
Supplement: Supplementary Information [file srep32980-s1.pdf]

Supplementary information for:

Title: Altered Intranetwork and Internetwork Functional Connectivity in Type 2 Diabetes Mellitus With and Without Cognitive Impairment

Authors: Shi-Qi Yang[1,*], Zhi-Peng Xu[2,6,*], Ying Xiong[1], Ya-Feng Zhan[3,4], Lin-Ying Guo[1], Shun Zhang[1], Ri-Feng Jiang[1], Yi-Hao Yao[1], Yuan-Yuan Qin[1], Jian-Zhi Wang[2], Yong Liu[4,5] & Wen-Zhen Zhu[1]

[1]Department of Radiology, Tongji Hospital, Tongji Medical College, Huazhong University of Science and Technology, Wuhan, Hubei, 430030, China.

[2]Department of Pathophysiology, School of Basic Medicine and the Collaborative Innovation Center for Brain Science, Key Laboratory of Ministry of Education of China for Neurological Disorders, Tongji Medical College, Huazhong University of Science and Technology, Wuhan, Hubei, 430030, China.

[3]School of Biomedical Engineering, Southern Medical University, Guangzhou, Guangdong, 510515, China.

[4]Brainnetome Center, Institute of Automation, Chinese Academy of Sciences, Beijing, 100190, China.

[5]National Laboratory of Pattern Recognition, Institute of Automation, Chinese Academy of Sciences, Beijing, 100190, China.

[6]Department of Neurology, Wuhan General Hospital of Guangzhou Command, Wuhan, Hubei, 430070, China.

*These authors contributed equally to this work.

Correspondence and requests for materials should be addressed to W.Z. (email: zhuwenzhen@hotmail.com) or Y.L. (email: yliu@nlpr.ia.ac.cn) or J.W. (email: wangjz@mail.hust.edu.cn)

Supplementary information:

Tables S1–S4

Table S1. Information of 36 prior defined ROIs

No.	ROI	RSN	MNI coordinates		
1	Posterior cingulate cortex (PCC)	DMN	0	-51	29
2	Medial prefrontal cortex (mPFC)	DMN	0	61	22
3	Left lateral parietal (lLP)	DMN	-48	-66	34
4	Right lateral parietal (rLP)	DMN	53	-61	35
5	Left inferior temporal (liTmp)	DMN	-65	-22	-9
6	Right inferior temporal (riTmp)	DMN	61	-21	-12
7	Medial thalamus (mdThal)	DMN	0	-9	7
8	Left posterior cerebellum (lpCBLM)	DMN	-28	-82	-32
9	Right posterior cerebellum (rpCBLM)	DMN	26	-89	-34
10	Left front eye field (lFEF)	DAN	-29	-5	55
11	Right front eye field (rFEF)	DAN	31	-5	54
12	Left posterior intraparietal sulcus (lpIPS)	DAN	-26	-65	52
13	Right posterior intraparietal sulcus (rpIPS)	DAN	28	-65	51
14	Left anterior intraparietal sulcus (laIPS)	DAN	-45	-37	48
15	Right anterior intraparietal sulcus (raIPS)	DAN	43	-36	46
16	Left middle temporal gyrus (lMT)	DAN	-52	-66	-4
17	Right middle temporal gyrus (rMT)	DAN	55	-62	-7
18	Dorsal mPFC (dmPFC)	CON	1	30	44
19	Left anterior PFC (laPFC)	CON	-45	50	-5
20	Right anterior PFC (raPFC)	CON	46	51	-7
21	Left superior parietal (lSP)	CON	-51	-50	49
22	Right superior parietal (rSP)	CON	53	-49	47
23	Right anterior cingulate cortex (rACC)	SAL	12	32	30
24	Left anterior cingulate cortex (lACC)	SAL	-13	34	16
25	Right ventral anterior cingulate cortex (rvACC)	SAL	10	34	-6
26	Left putamen (lPut)	SAL	-19	3	9
27	Right putamen (rPut)	SAL	25.5	18	8
28	Left insula (lIns)	SAL	-42	6	4
29	Right insula (rIns)	SAL	43	7	2
30	Left motor cortex (lMC)	SMN	-40	-23	53
31	Right motor cortex (rMC)	SMN	41	-22	48
32	Supplemental motor area (SMA)	SMN	1	-18	49
33	Left primary visual (lV1)	SMN	-8	-83	0
34	Right primary visual (rV1)	SMN	7	-83	0
35	Left primary auditory (lA1)	SMN	-64	-28	13
36	Right primary auditory (rA1)	SMN	62	-24	13

Table S2. Intergroup differences and clinical correlations at the integrity level

Nodename	lpCBLM	rpCBLM	rIns
MeaHC	18.53	15.54	15.83
SeHC	0.55	0.44	0.6
MeanDMCN	16.99	14.06	14.67
SeDMCN	0.43	0.42	0.6
MeanDMCI	17.03	14.12	13.82
SeDMCI	0.55	0.44	0.6
Anova_F	3.791	4.036	3.58
Anova_P	0.029	0.023	0.035
RandPermutationP	0.029	0.024	0.035
HC-DMCN	0.035	0.021	0.179
HC-DMCI	0.027	0.021	0.007
DMCI-DMCN	0.95	0.911	0.236
MMSE_Cor_DMCN	0.121	-0.163	-0.049
MMSE_P_DMCN	0.621	0.505	0.843
MMSE_Cor_DMCI	-0.093	-0.223	0.097
MMSE_P_DMCI	0.704	0.36	0.691
MoCA_Cor_DMCN	0.224	-0.052	-0.171
MoCA_P_DMCN	0.357	0.833	0.484
MoCA_Cor_DMCI	-0.026	0.051	0.082
MoCA_P_DMCI	0.915	0.834	0.739
HbA1c_Cor_DMCN	-0.398	-0.037	-0.022
HbA1c_P_DMCN	0.091	0.881	0.928
HbA1c_Cor_DMCI	-0.164	-0.19	0.159
HbA1c_P_DMCI	0.501	0.436	0.515
Duration_Cor_DMCN	-0.445	0.071	-0.245
Duration_P_DMCN	0.056	0.772	0.312
Duration_Cor_DMCI	-0.359	-0.501	0.341
Duration_P_DMCI	0.131	0.029	0.153

Table S3. Intergroup differences and clinical correlations at the network level (intranetwork)

Nodename	DMN	CON
MeanHC	0.77	0.64
SeHC	0.02	0.02
MeanDMCN	0.73	0.63
SeDMCN	0.02	0.03
MeanDMCI	0.7	0.55
SeDMCI	0.02	0.02
Anova_F	3.465	4.406
Anova_P	0.038	0.017
RandPermutationP	0.042	0.017
HC-DMCN	0.116	0.982
HC-DMCI	0.02	0.008
DMCI-DMCN	0.28	0.017
MMSE_Cor_DMCN	0.016	-0.07
MMSE_P_DMCN	0.949	0.775
MMSE_Cor_DMCI	0.251	0.598
MMSE_P_DMCI	0.3	0.007
MoCA_Cor_DMCN	0.101	-0.475
MoCA_P_DMCN	0.681	0.04
MoCA_Cor_DMCI	0.321	0.297
MoCA_P_DMCI	0.181	0.218
HbA1c_Cor_DMCN	0.179	0.18
HbA1c_P_DMCN	0.462	0.461
HbA1c_Cor_DMCI	-0.306	0.209
HbA1c_P_DMCI	0.203	0.391
Duration_Cor_DMCN	-0.076	0.361
Duration_P_DMCN	0.757	0.128
Duration_Cor_DMCI	-0.404	0.48
Duration_P_DMCI	0.086	0.038

Table S4. Intergroup differences and clinical correlations at the connectivity level

Nodename	Nodename	MeanHC	SeHC	MeanDMCN	SeDMCN	MeanDMCI	SeDMCI	Anova_F	Anova_P	RandPermutationP	HC-DMCN	HC-DMCI	DMCI-DMCN
laPFC	lSP	0.69	0.03	0.66	0.04	0.48	0.03	10.837	0	0	0.624	0	0.001
rIns	rV1	0.42	0.03	0.28	0.03	0.29	0.03	7.223	0.002	0.002	0.004	0.003	0.748
rACC	rvACC	0.53	0.03	0.51	0.03	0.38	0.03	7.239	0.002	0.002	0.589	0	0.004
laPFC	rSP	0.69	0.04	0.66	0.03	0.54	0.04	6.44	0.003	0.003	0.595	0.002	0.004
lLP	lA1	0.31	0.02	0.24	0.02	0.36	0.02	5.981	0.005	0.004	0.038	0.171	0.002
lFEF	lPut	0.65	0.03	0.77	0.03	0.66	0.03	5.897	0.005	0.005	0.005	0.839	0.008
raIPS	rIns	0.56	0.04	0.54	0.04	0.42	0.04	5.623	0.006	0.006	0.753	0.002	0.006
rV1	lA1	0.47	0.03	0.42	0.04	0.57	0.03	5.292	0.008	0.006	0.257	0.032	0.004
rIns	lV1	0.39	0.04	0.26	0.03	0.27	0.04	5.024	0.01	0.008	0.015	0.012	0.786
raIPS	lPut	0.54	0.02	0.59	0.03	0.46	0.02	5.052	0.01	0.01	0.249	0.039	0.005
liTmp	rpCBLM	1.04	0.03	0.89	0.03	0.97	0.03	5.124	0.009	0.01	0.003	0.14	0.093
rFEF	lPut	0.72	0.02	0.82	0.03	0.7	0.02	4.869	0.011	0.011	0.009	0.675	0.012
liTmp	riTmp	0.51	0.04	0.54	0.04	0.38	0.04	4.888	0.011	0.012	0.595	0.025	0.006
mPFC	rpCBLM	0.87	0.04	0.74	0.04	0.71	0.04	4.835	0.012	0.012	0.035	0.007	0.546
rIns	SMA	0.84	0.04	0.87	0.03	0.75	0.04	4.548	0.015	0.013	0.488	0.048	0.001
rIns	lMC	0.65	0.04	0.63	0.03	0.53	0.04	4.356	0.018	0.014	0.733	0.01	0.012
PCC	mdThal	0.44	0.03	0.48	0.03	0.36	0.03	4.579	0.015	0.014	0.289	0.079	0.003
PCC	lLP	0.45	0.03	0.43	0.03	0.32	0.03	4.781	0.012	0.014	0.762	0.011	0.023
lFEF	rpIPS	0.57	0.04	0.66	0.03	0.51	0.04	4.654	0.014	0.015	0.068	0.231	0.004
lMT	rIns	0.53	0.04	0.53	0.04	0.4	0.04	4.528	0.015	0.018	0.976	0.012	0.005
dmPFC	rvACC	0.3	0.03	0.29	0.02	0.21	0.03	4.116	0.022	0.019	0.743	0.012	0.022
lIns	lV1	0.49	0.03	0.37	0.04	0.43	0.03	4.189	0.02	0.02	0.014	0.108	0.152
rvACC	rMC	0.57	0.02	0.59	0.03	0.67	0.02	4.15	0.021	0.022	0.397	0.01	0.077
laIPS	rIns	0.71	0.04	0.62	0.03	0.59	0.04	3.971	0.025	0.023	0.078	0.009	0.46
rFEF	rIns	0.69	0.03	0.73	0.04	0.59	0.03	4.01	0.024	0.023	0.538	0.027	0.013
rpCBLM	rA1	0.32	0.03	0.22	0.02	0.29	0.03	3.765	0.029	0.026	0.013	0.414	0.059
lPut	rA1	0.33	0.02	0.21	0.04	0.26	0.02	3.865	0.027	0.026	0.01	0.106	0.231
lFEF	lMC	0.3	0.03	0.19	0.03	0.3	0.03	3.9	0.026	0.026	0.011	0.975	0.026
lMT	raPFC	0.44	0.03	0.34	0.03	0.34	0.03	3.87	0.027	0.029	0.027	0.023	0.932
lpCBLM	laPFC	0.96	0.04	0.85	0.03	0.87	0.04	3.846	0.027	0.03	0.021	0.047	0.577
lIns	rV1	0.56	0.03	0.45	0.03	0.5	0.03	3.76	0.03	0.031	0.013	0.165	0.17
rpIPS	laPFC	0.92	0.03	0.99	0.03	0.87	0.03	3.685	0.032	0.031	0.155	0.257	0.007
lSP	lIns	0.35	0.03	0.23	0.03	0.32	0.03	3.747	0.03	0.032	0.009	0.512	0.058
liTmp	rMT	0.4	0.04	0.4	0.04	0.28	0.04	3.699	0.031	0.033	0.963	0.023	0.016
lMT	rMT	0.68	0.03	0.72	0.03	0.6	0.03	3.669	0.032	0.033	0.391	0.087	0.014
rvACC	lMC	0.57	0.02	0.57	0.03	0.66	0.02	3.58	0.035	0.034	0.798	0.02	0.055
lLP	rA1	0.4	0.03	0.31	0.01	0.37	0.03	3.628	0.033	0.034	0.012	0.411	0.05
riTmp	rMT	0.38	0.04	0.39	0.04	0.27	0.04	3.46	0.039	0.036	0.821	0.033	0.015
lSP	lA1	0.47	0.04	0.34	0.03	0.41	0.04	3.387	0.041	0.037	0.016	0.26	0.145
PCC	rMT	0.37	0.03	0.4	0.03	0.29	0.03	3.373	0.042	0.039	0.532	0.057	0.014
lLP	rIns	0.11	0.02	0.06	0.02	0.12	0.02	3.38	0.041	0.04	0.046	0.761	0.014
rIns	rMC	0.53	0.04	0.46	0.04	0.4	0.04	3.445	0.039	0.04	0.205	0.012	0.19
lpCBLM	rA1	0.32	0.03	0.24	0.02	0.31	0.03	3.458	0.039	0.04	0.012	0.739	0.039
lFEF	rIns	0.55	0.03	0.57	0.04	0.46	0.03	3.402	0.041	0.04	0.578	0.048	0.017
lpCBLM	rPut	0.19	0.01	0.14	0.02	0.14	0.01	3.39	0.041	0.042	0.023	0.028	0.95
rvACC	lV1	0.45	0.02	0.51	0.03	0.55	0.02	3.499	0.037	0.043	0.122	0.008	0.324
lpCBLM	dmPFC	0.5	0.04	0.39	0.02	0.42	0.04	3.316	0.044	0.044	0.018	0.101	0.517
rMT	lA1	0.62	0.04	0.7	0.03	0.57	0.04	3.2	0.049	0.049	0.133	0.416	0.008
rSP	rvACC	0.23	0.03	0.22	0.02	0.16	0.03	3.188	0.049	0.05	0.786	0.024	0.032
mPFC	lSP	0.14	0.03	0.03	0.03	0.05	0.03	3.196	0.049	0.05	0.02	0.077	0.674

MMSE_Cor_DMCN	MMSE_P_DMCN	MMSE_Cor_DMCI	MMSE_P_DMCI	MoCA_Cor_DMCN	MoCA_P_DMCN	MoCA_Cor_DMCI	MoCA_P_DMCI
0.064	0.795	0.311	0.195	-0.339	0.156	-0.025	0.92
-0.36	0.13	0.142	0.563	-0.192	0.432	-0.014	0.954
0.25	0.302	0.212	0.383	-0.223	0.358	0.165	0.5
0.057	0.816	0.34	0.154	-0.207	0.395	0.202	0.407
0.325	0.175	-0.432	0.065	0.22	0.366	-0.505	0.027
0.396	0.094	0.262	0.279	-0.02	0.934	0.261	0.281
-0.409	0.082	0.12	0.625	-0.459	0.048	0.047	0.85
-0.215	0.377	0.169	0.488	0.001	0.995	0.482	0.036
-0.45	0.053	-0.039	0.872	-0.101	0.68	0.043	0.863
0.501	0.029	-0.06	0.807	0.106	0.664	0.025	0.918
0.176	0.471	-0.037	0.881	0.14	0.567	0.089	0.716
0.438	0.06	0.177	0.469	-0.044	0.858	0.347	0.145
0.116	0.638	0.355	0.136	-0.098	0.69	0.352	0.14
-0.03	0.904	0.345	0.148	0.086	0.727	0.376	0.113
-0.247	0.309	-0.05	0.839	-0.074	0.762	0.006	0.982
-0.408	0.083	-0.082	0.74	-0.238	0.327	0.166	0.498
-0.368	0.122	-0.503	0.028	0.327	0.172	-0.266	0.27
-0.237	0.329	0.12	0.625	-0.174	0.477	0.266	0.271
0.01	0.967	0.258	0.287	0.243	0.316	0.341	0.153
-0.143	0.56	0.029	0.905	-0.215	0.377	0.093	0.706
0.432	0.064	0.391	0.098	0.086	0.726	0.331	0.166
-0.376	0.113	0.003	0.99	-0.009	0.97	0.275	0.254
0.191	0.432	0.478	0.039	0.463	0.046	0.329	0.168
-0.474	0.04	0.296	0.219	-0.273	0.258	0.167	0.494
-0.173	0.478	0.306	0.202	-0.201	0.408	0.261	0.28
-0.124	0.613	-0.41	0.081	-0.014	0.956	-0.333	0.164
-0.068	0.783	-0.314	0.19	-0.331	0.166	-0.102	0.678
0.043	0.862	0.089	0.716	-0.12	0.626	0.045	0.855
0.247	0.308	0.459	0.048	0.384	0.104	0.369	0.12
-0.222	0.36	0.04	0.872	0.112	0.649	-0.313	0.192
-0.223	0.359	0.308	0.199	-0.034	0.891	0.388	0.1
0.215	0.377	0.056	0.821	-0.241	0.321	0.238	0.326
-0.14	0.568	-0.396	0.094	-0.195	0.424	-0.112	0.648
0.345	0.148	-0.477	0.039	0.15	0.54	-0.466	0.045
-0.286	0.236	-0.053	0.829	0.136	0.578	0.117	0.633
0.397	0.093	0.315	0.19	0.288	0.231	0.359	0.131
0.133	0.588	-0.239	0.325	0.246	0.309	-0.155	0.526
0.301	0.21	-0.105	0.668	-0.26	0.283	-0.312	0.193
-0.447	0.055	-0.693	0.001	-0.264	0.275	-0.357	0.134
0.51	0.026	-0.263	0.277	0	0.999	-0.3	0.213
0.325	0.174	-0.031	0.9	-0.371	0.118	-0.168	0.493
-0.323	0.177	-0.076	0.759	-0.135	0.582	0.106	0.666
-0.304	0.206	-0.19	0.435	-0.085	0.731	-0.234	0.334
-0.129	0.599	0.144	0.556	-0.329	0.169	0.258	0.287
-0.032	0.895	-0.025	0.919	-0.118	0.629	0.248	0.306
0.104	0.672	0.183	0.453	0.151	0.538	-0.03	0.903
-0.086	0.727	0.292	0.225	0.184	0.451	0.295	0.221
-0.091	0.712	-0.519	0.023	0.127	0.604	-0.335	0.161
0.222	0.361	0.251	0.3	0.273	0.258	0.132	0.59
0.197	0.418	0.111	0.651	-0.247	0.309	0.171	0.485

HbA1c_Cor_DMCN	HbA1c_P_DMCN	HbA1c_Cor_DMCI	HbA1c_P_DMCI	Duration_Cor_DMCN	Duration_P_DMCN	Duration_Cor_DMCI	Duration_P_DMCI
0.045	0.854	0.247	0.309	0.009	0.972	0.424	0.071
0.056	0.821	0.228	0.347	0.116	0.636	0.082	0.739
-0.314	0.191	0.222	0.361	0.034	0.892	0.153	0.533
0.076	0.758	0.055	0.824	0.046	0.85	0.218	0.371
0.02	0.935	0.422	0.072	0.034	0.889	0.351	0.14
-0.058	0.812	-0.042	0.863	0.122	0.62	-0.302	0.209
0.031	0.899	0.03	0.904	-0.215	0.376	0.196	0.422
-0.094	0.703	-0.045	0.853	-0.185	0.448	-0.26	0.281
0.133	0.588	0.044	0.858	0.127	0.606	-0.026	0.914
-0.387	0.101	-0.17	0.486	-0.111	0.652	-0.208	0.394
0.389	0.1	-0.163	0.505	0.092	0.707	0.017	0.946
-0.445	0.056	-0.249	0.305	0.114	0.641	-0.226	0.351
0.06	0.808	0.1	0.684	0.033	0.894	0.383	0.105
-0.019	0.938	-0.506	0.027	-0.085	0.729	-0.354	0.137
0.271	0.261	0.006	0.98	0.089	0.716	0.194	0.427
0.246	0.31	0.087	0.722	-0.061	0.803	-0.016	0.947
0.037	0.881	-0.109	0.658	-0.224	0.356	-0.345	0.148
-0.025	0.918	-0.501	0.029	0.257	0.288	-0.529	0.02
0.154	0.53	0.215	0.377	-0.188	0.442	0.056	0.819
-0.103	0.675	0.018	0.94	-0.165	0.5	0.107	0.663
-0.427	0.068	-0.004	0.986	0.088	0.72	0.227	0.35
0.019	0.937	-0.258	0.287	-0.098	0.69	-0.349	0.144
-0.146	0.551	0.019	0.94	0.079	0.749	0.18	0.461
0.089	0.717	-0.144	0.557	-0.2	0.412	0.183	0.453
0.1	0.684	-0.044	0.86	-0.108	0.66	0.262	0.278
0.093	0.704	0.276	0.253	-0.071	0.773	0.106	0.667
-0.066	0.789	0.023	0.925	0.128	0.603	-0.246	0.309
0.013	0.957	0.165	0.499	0.003	0.99	0.013	0.959
-0.161	0.509	-0.197	0.418	-0.055	0.822	0.035	0.887
0.158	0.518	0.144	0.556	-0.183	0.454	0.137	0.577
-0.15	0.539	-0.004	0.986	-0.196	0.42	-0.156	0.524
-0.35	0.142	-0.203	0.404	0.005	0.984	-0.321	0.18
0.389	0.1	-0.427	0.068	0.319	0.182	-0.293	0.223
-0.201	0.41	-0.034	0.89	0.076	0.759	-0.084	0.732
-0.514	0.024	-0.339	0.156	-0.422	0.072	-0.521	0.022
-0.281	0.245	-0.03	0.903	0.25	0.301	-0.007	0.977
0.026	0.914	0.395	0.094	0.146	0.552	0.312	0.194
-0.195	0.423	-0.146	0.551	0.127	0.603	0.191	0.433
0.037	0.879	0.193	0.429	-0.237	0.329	0.093	0.705
-0.46	0.048	-0.222	0.361	0.036	0.882	-0.187	0.443
0.02	0.934	0.411	0.081	0.059	0.809	0.48	0.038
0.267	0.27	-0.268	0.268	-0.011	0.964	-0.128	0.6
-0.066	0.788	0.199	0.415	-0.181	0.459	0.303	0.207
0.078	0.752	-0.084	0.731	0.036	0.884	0.092	0.707
-0.433	0.064	-0.266	0.271	0.166	0.498	-0.32	0.182
-0.087	0.723	-0.15	0.541	0.066	0.788	0.091	0.71
-0.477	0.039	0.048	0.847	-0.037	0.882	0.037	0.882
0.24	0.323	-0.323	0.178	-0.282	0.241	-0.302	0.209
-0.23	0.344	-0.042	0.865	0.098	0.689	-0.072	0.769
-0.288	0.232	-0.166	0.498	0.293	0.223	-0.427	0.068
